# Cognitive and physical impact of combined exercise and cognitive intervention in older adults with mild cognitive impairment: A systematic review and meta-analysis

**DOI:** 10.1371/journal.pone.0308466

**Published:** 2024-10-03

**Authors:** Qing Yi, Zuhong Liu, Fei Zhong, Victor Selvarajah Selvanayagam, Jadeera Phaik Geok Cheong

**Affiliations:** 1 Faculty of Sports and Exercise Science, Universiti Malaya, Kuala Lumpur, Malaysia; 2 Department of Physical Education, Shanghai Sanda University, Shanghai, China; 3 Department of Sports and Exercise Science, Zhejiang University, Zhejiang, China; Università degli Studi di Milano: Universita degli Studi di Milano, ITALY

## Abstract

Emerging studies have examined the effectiveness of combined exercise and cognitive intervention (combined intervention) on the cognitive function of older adults with MCI, but the conclusions remain disputed. Our study aimed to comprehensively examine the efficacy of the combined intervention on cognitive and physical function in older adults with MCI. PubMed, Cochrane Library, EMBASE, and PsycINFO were retrieved to identify the relevant articles. Twelve eligible studies were included, and the results showed that combined intervention significantly improved global cognition SMD = 0.26, 95% CI [0.14-0.39], *p*<0.0001), executive function (SMD = 0.40, 95% CI [0.25-0.56], *p* < 0.00001), memory (SMD = 0.30, 95% CI [0.22-0.39], *p* <0.00001), and gait performance (SMD = 0.32, 95% CI [0.03-0.62], *P* = 0.03) compared to the control group. Combined intervention significantly improved executive function compared to single exercise intervention while not showing a statistically significant difference compared to single cognitive intervention. Moreover, no significant difference was observed between simultaneously and sequentially combined intervention. The finding indicated that combined intervention is efficacious in improving global cognition and selectively enhancing cognitive domains and physical function in older adults with MCI. More research with robust designs should be conducted, particularly involving comparisons with single interventions and different types of combined interventions.

## Introduction

It was predicted that there would be 65.7 million individuals with dementia by 2030 [1], which will lead to considerable effects on the socio-economic system [2]. Mild cognitive impairment (MCI) is an intermediate stage in the transition from normal cognitive decline to dementia [3], and it progresses more rapidly than expected without affecting daily function [4]. The 10% yearly conversion rate from MCI to dementia is much higher than the 1–2% incidence rate for the general population [5]. Therefore, MCI offers the ideal window to delay the onset of dementia [6].

To achieve additive effects, multimodal intervention strategies, particularly the combination of exercise intervention and cognitive intervention, have garnered attention [7]. Accumulating evidence suggests that combined intervention can provide additional health and functional benefits to individuals with MCI [8–10]. Combined intervention can be classified [11] as follows: (1) simultaneous combination, where exercise and cognitive intervention are performed concurrently in dual-tasking or exergaming format; (2) sequential combination, where exercise and cognitive intervention are separate and can be done on the same or separate days. Most meta-analyses reported that combined intervention could improve cognition when compared to the control group [10, 11], single exercise intervention [12] and cognitive intervention [10, 12]. However, some studies indicated that the cognitive benefits of combined intervention are not superior to those of single exercise or cognitive interventions [13, 14]. Furthermore, there is a paucity of research examining the efficacy of these two categories of combined intervention [13, 15] and their effects on physical function [16].

Previous meta-analyses seldom restricted the components of exercise intervention in combined interventions, which might encompass aerobic, resistance, or multicomponent exercises. Nevertheless, incorporating diverse exercise components may increase heterogeneity. Notable, only one review restricted the type of exercise intervention to aerobic exercise, investigating the efficacy of combined aerobic exercise with cognitive training on cognition in stroke patients [17]. Multicomponent exercise could combine different training programs (e.g., resistance, aerobic, and balance training) in one training session to develop multiple functions simultaneously without prolonged training time [18], which is the most recommended exercise for older adults [19]. However, no study limited the type of exercise intervention to multicomponent exercises.

This study examines the effectiveness of the combined multicomponent exercises and cognitive intervention on cognitive and physical function among older adults with MCI. The objectives are as follows: (1) to evaluate the efficacy of combined intervention to the control, single exercise, and cognitive intervention group; (2) to compare the effectiveness of simultaneously and sequentially combined intervention.

## Method

This meta-analysis complied with the Preferred Reporting Items for Systematic Reviews and Meta Analyses (PRISMA) guidelines [20] and PRISMA extension for meta-analysis [21]. Our study has been registered on the International Platform of Registered Systematic Review and Meta-analysis Protocols (INPLASY, registration number: INPLASY2022110121; https://inplasy.com/inplasy-2022-11-0121/).

### Search and selection strategies

PubMed, Embase, Cochrane Library, and PsycINFO databases were searched on February 7, 2023, for relevant studies. A second search was performed on October 25, 2023, to reduce database update bias. No restrictions were set on the publication date or type. To enhance the comprehensiveness of the search terms, we consulted previous studies [11, 13]. Search strategies were finalized using the Boolean operators "OR" and "AND," along with terms such as "exercise intervention," "cognitive intervention," "combination," "aging," and "MCI." The specific search strategies can be found in supplementary material (S1 Appendix). Additionally, to ensure completeness, we manually searched the references of the included publications.

Documents were stored, categorized, and removed using EndNote 20. Initially, two researchers (Qing Yi and Zuhong Liu) independently screened the titles and abstracts of the literature. Subsequently, the full text of the selected documents was carefully read to identify the eventually eligible literature. Disagreements in literature selection were resolved through discussion between the two researchers until a consensus was reached.

### Selection criteria

#### Types of studies

Published randomized controlled trials (RCTs) examining the effectiveness of the combined intervention on cognitive or physical performance in MCI patients were included. Case reports, reviews, study protocols, and case series were excluded.

#### Types of participants

The participants in eligible studies should include MCI patients aged 60 years or older, encompassing both amnestic MCI and non-amnestic MCI. In addition, the participants included in the study should not have other types of diseases.

#### Types of interventions

The exercise intervention scheme should be a combined intervention, limiting the exercise intervention component to multicomponent exercise, and not limiting the component of cognitive intervention. The intervention schemes should be structured and should include components such as the contents, the dose, and the setting.

#### Types of comparisons

The comparisons need to meet one of the following conditions: (1) a single exercise intervention group; (2) a single cognitive intervention group; (3) a control group, either an active control (e.g., health education, leisure activities, or toning exercises) or an inactive control (e.g., no-contact, waiting list, or business as usual).

#### Types of outcomes

The outcomes should encompass at least one measure of cognitive function (either global or specific cognition domains) or physical function (such as mobility and balance). Additionally, the outcomes should measure the change from baseline to post-intervention.

### Data extraction

The data were extracted by the two researchers separately using a self-created standardized form. Disagreements will be further discussed by the two researchers until a consensus is reached. The author’s name, publication year, nation, sample size, age, types of intervention, length of intervention, frequency of intervention, types of control, and outcomes were retrieved from the literature.

### Quality assessment

The two researchers independently assessed the quality of the included articles using the Cochrane Collaboration Risk of Bias Tool [22]. If any disputes are encountered during the quality assessment, the two researchers will discuss the dispute until an agreement is reached. The criteria for "blinding of participants and personnel (performance bias)" were excluded from the risk assessment because exercise and cognitive interventions do not allow participant blinding. Therefore, a qualitative assessment was conducted on six aspects of the RCTs: random sequence generation (selection bias), blinding of outcome assessment (detection bias), allocation concealment (selection bias), incomplete outcome data (attrition bias), selective reporting (reporting bias), and other biases. Each index was classified as "high risk," "low risk," or "unclear risk."

### Statistical analysis

Review Manager 5.4 was employed for data analysis in this study. The specific analysis process encompassed reporting the combined effect size and heterogeneity, as well as generating and exporting the forest plots. The outcomes in the encompassed literature (cognitive function and physical function) constituted continuous variables. As the data were amalgamated from studies utilizing inconsistent scales but measuring the same outcome, the Standardized Mean Difference (SMD) was chosen as the effect indicator, with a 95% confidence interval (CI) provided. The pooled SMDs represented the effect size for each outcome, including global cognition, specific cognitive domains, and physical function.

If I^2^ >50%, the random-effects model was adopted instead of the fixed-effects model [23]. Moreover, heterogeneity tests were performed using the statistic I^2^. Heterogeneity levels were categorized as low (25%), moderate (50%), and high (75%) [24]. In cases where I2 > 50%, sensitivity analysis was executed [24]. Given the limited number of eligible articles, the present study refrained from undertaking the funnel plot asymmetry examination to investigate the publication bias [25].

## Results

### Study selection

**Fig 1:**
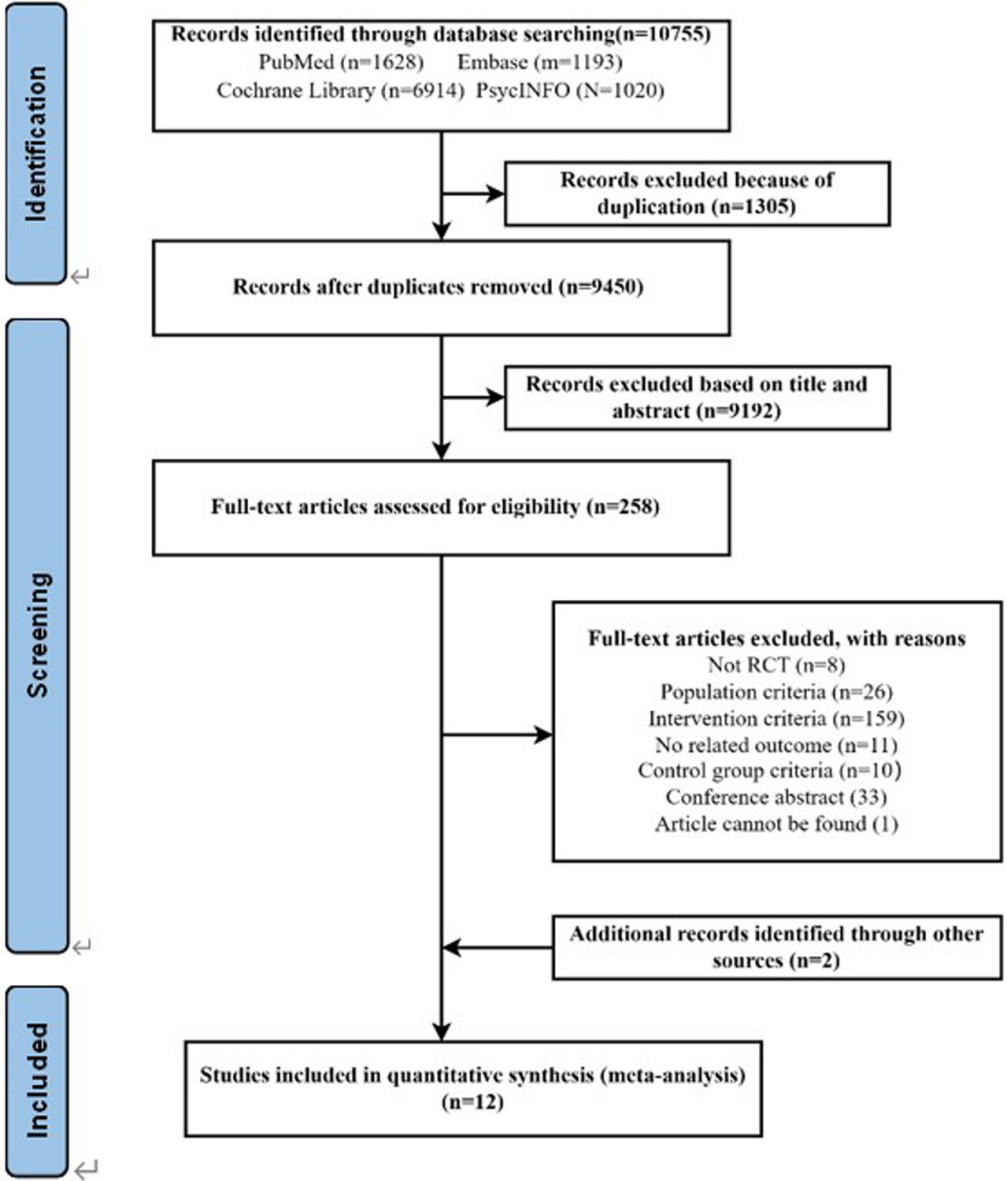
Flowchart of literature retrieval and screening.

The initial retrieval found 10,755 records based on a pre-determined search strategy. After removing 1,305 duplicate documents, we excluded 9,192 articles out of 9,450 by screening the titles and abstracts. Subsequently, a full-text screening was performed on 258 articles, and this process eliminated 248 articles that did not match the requirements. Of these, 10 unique studies met the inclusion criteria. We also identified two additional eligible articles by searching the references of included articles. Therefore, 12 articles were finally included in our study (Fig 1).

### Study characteristics

As shown in Table 1, the eligible articles involved 1,075 participants, ranging from 17 to 308, and ages 60.0 to 93.0 years. Those interventions took place between one to three times/week, with session lengths ranging from 18 to 120 minutes, and the intervention durations varied from six weeks to three years. Regarding the multicomponent exercise component, five studies [26–30] involved aerobic, strength, and balance exercise; two studies [31, 32] incorporated both aerobic and balance exercises; two studies [33, 34] involved aerobic and resistance exercises; two studies [35, 36] consisted of strength, aerobic, flexibility, and balance exercises, and one study [37] included strength and balance exercises. Regarding the combined methods, five studies [26, 27, 31, 34, 35] used sequential design, and four studies [1–3, 28, 32, 36, 37] used a simultaneous design. Additionally, three studies [29, 30, 33] included both simultaneous and sequential combinations. Regarding the comparison condition, seven studies [26–28, 31, 32, 35, 37] used a control group, and two studies [34, 36] used three comparisons including control, exercise intervention, and cognitive intervention alone. For the outcomes, five studies [28–31, 37] included both cognitive and physical function outcomes, six studies [26, 27, 32–35] focused only on cognitive function outcomes, and one article [36] examined only physical function outcomes.

**Table 1:** Basic information of eligible literature.

Author/ Year /Country	Participants Characteristics			Combined Intervention Design				Comparisons	Outcomes
	Size	Mean Age	Female (%)	Exercise Intervention Components	Cognitive Intervention Components	Combination Method	Session Length (min); Session /Week; Duration		
Lipardo et al. 2020 [36];Hong Kong	92	69.00±8.30	79.00%	BT; ST; ET; FT	Executive; Orientation; Attention; Memory training.	simultaneous	60–90 min/session; 3 sessions/week; 12 weeks	EI; CIT; CG	PF
Poptsi et al. 2022 [35]; Greek	283	CI:68.12±6.31; CG:67.11±9.10	81.20%	AT; ST; FT; BT	Longitudinal Multicomponent Cognitive Training program.	Sequential	120 min/session; 1session/week; 3 years	CG	CF
Suzuki et al. 2013 [27]; Japan	92	75.40±67.10	44.49%	AT; ST; BT	Inventing their own poem, etc.	Sequential	90 min/session; 2 sessions/week; 24 weeks	CG	CF
Park et al. 2017 [33]; Korea	21	Over 60	71.43%	AT; ST	Count the number; Naming of pictures (Flowers); Count the number; Naming of pictures (Fruits), etc.	Simultaneous; Sequential	60 min/session; 2 sessions/week; 8 weeks	Sequentially CI	CF
Shimada et al. 2018 [28]; Japan	308	CI:71.60±5.00;CG:71.60±4.90	50.00%	AT; ST; BT	Played word games, etc.	Simultaneous	90min/session; 1 session/week; 40 weeks	CG	CF; PF
Liao et al. 2019 [30]; Taiwan, China	34	CI (CPC): 73.10±6.80; CI (VR): 75.50±5.20	CI:61.11%; VRG:75.00%	1. CPC group: ST; AT; BT 2.VR group: tai chi; AT; ST	1. CPC group: reciting poems; enumerating animals or flow; practicing math calculations. 2. VR group: VR game.	Simultaneous; Sequential	60 min/session; 3 sessions/week; 12 weeks	Sequentially CI	CF; PF
Liao et al. 2020 [29]; Taiwan, China	34	CI (CPC): 73.10±6.80; CI (VRG): 75.50±5.20	CI:61.11%; VR:75.00%	1. CPC group: ST; AT; BT 2.VR group: tai chi; AT; ST	1. CPC group: reciting poems; enumerating animals or flow; practicing math calculations. 2. VR group: VR game.	Simultaneous; Sequential	60 min/session; 3 sessions/week; 12 weeks	Sequentially CI	PF; CF
Park et al. 2019 [31]; Korea	45	IG: 70.55±6.46; CG:72.76 ±5.37	CI:68.00%; CG:70.83%	AT; BT	Word games; Numerical mcalculations; Memory span games, etc.	Sequential	110 min/session; 1 session/week; 24 weeks	CG	CF; PF
Damirchi et al. 2018 [34]; Iran	44	EI:68.81;CIT:67.90; CI:67.76; CG:69.11	NC	AT; ST	"Modified My Better Mind" program.	Sequential	30-120min; 3 sessions/week; 8 weeks.	EI; CIT; CG	CF
Suzuki et al. 2012 [26]; Japan	47	75.00	46.00%	ST; AT; BT	Invent their own poem; memorized a step pattern inconsecutive square sements.	Sequential	90 min/session; 2 sessions/week; 48 weeks	CG	CF
Delbroek et al 2017 [37]; Belgium	17	87.20 ±5.96	65.00%	ST; BT	Memory; Attention; Dual tasking.	Simultaneous	18–30 min/session; 2 sessions/week; 6 weeks	CG	CF; PF
Kounti et al. 2011 [32]; Greek	58	CI:70.48±7.29; CG:67.83±7.52	79.31%	AT; BT	Visual; Verbal.	Simultaneous	90 min/session; 1 session/week; 20 weeks	CG	CF

Note: CI, combined intervention; CG, control group; CF, cognitive function; PF, physical function; EI, exercise intervention; CIT, cognitive intervention; AE, Aerobic training; ST, Strength training: BT, Balance training; CPC, Combined Physical and Cognitive Training; NC, not clear; VR game, Virtual Reality-Based Physical and Cognitive Training.

### Risk of bias of included studies

**Fig 2:**
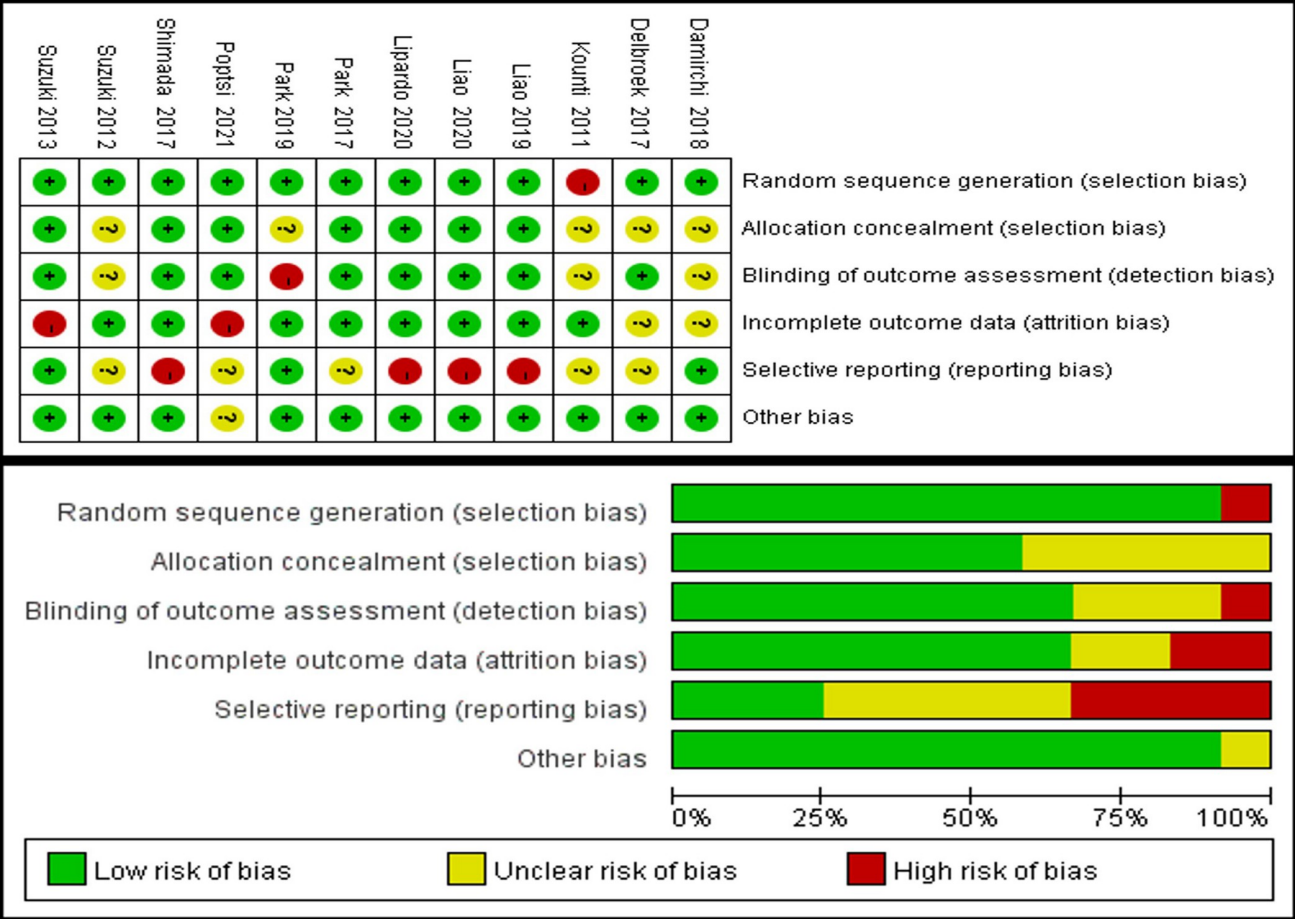
Risk of bias evaluation.

As shown in Fig 2, the graph utilizes green, yellow, and red colors to represent low risk, unclear risk, and high risk, respectively [24]. Regarding random sequence generation, eleven studies [26–31, 33–37] were assessed as low risk, while one study, while one study [32] was deemed high risk. The risk of allocation concealment was determined to be low in seven studies [27–30, 33, 35, 36] and unclear in five studies [26, 31, 32, 34, 37]. Eight studies [27–30, 33, 35–37] were classified as low risk, three studies [26, 32, 34] as unclear risk, and one study [31] as high risk for blinding of outcome assessment. Eight studies [26, 28–33, 36] were categorized as low risk, two studies [34, 37] as unclear, and two studies [27, 35] as high risk for incomplete outcome data. Additionally, three studies [27, 31, 34] were determined to be low risk, five studies [26, 32, 33, 35, 37] as unclear risk, and four studies [28–30, 36] was high risk for selective reporting. The intervention setting was considered another potential source of bias. In eleven studies [26–34, 36, 37], the training process was supervised by therapists or researchers, while one study during the intervention, and one study [35] did not provide relevant information.

### Effects of combined intervention versus control group

#### Cognitive function

**Fig 3:**
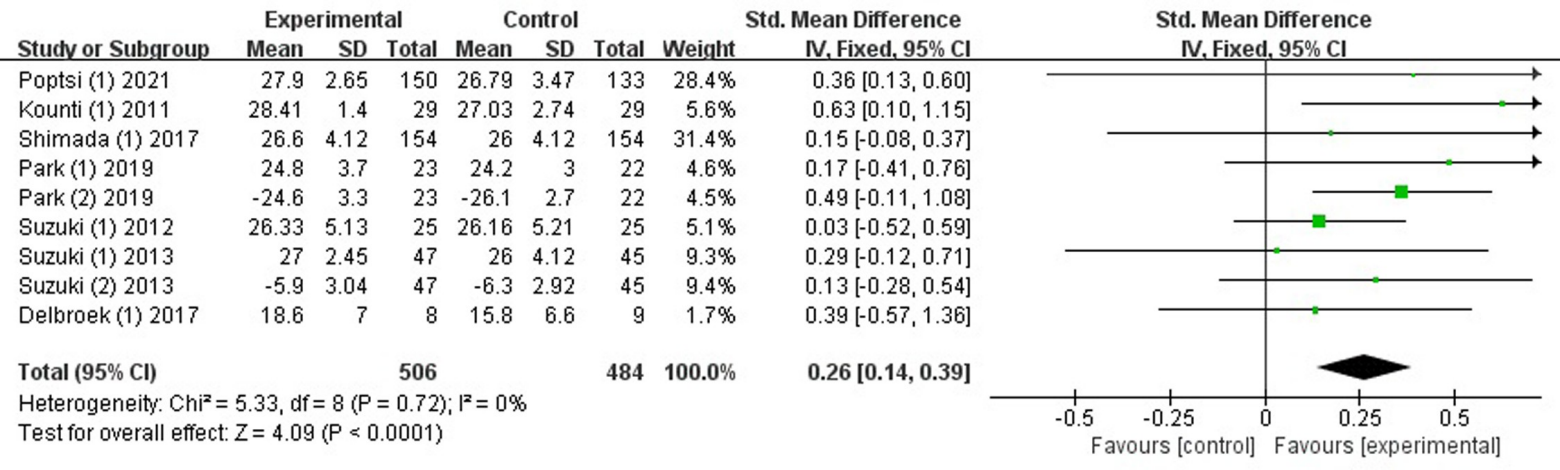
Forest plot of global cognition.

*Global cognition*. Seven studies [26–28, 31, 32, 35, 37] evaluated the effectiveness of the combined intervention on global cognition. Owing to the different assessment tools used across studies, this analysis used the Standard Mean Difference (SMD) and fixed-effects (FE) model. The results demonstrates that combined intervention significantly enhanced global cognition, with low heterogeneity (SMD = 0.26, 95% CI [0.14-0.39], *p*<0.0001, I^2^ = 0%, Fig 3).

**Fig 4:**
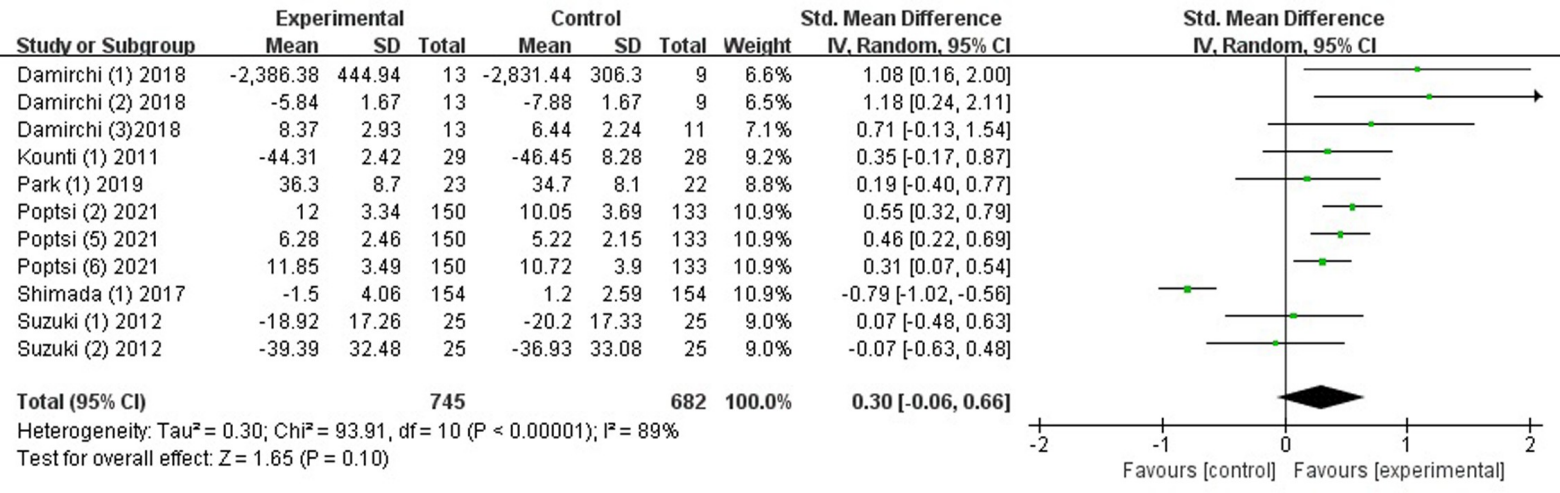
Forest plot of executive function.

*Executive function*. Six studies [26, 28, 31, 32, 34, 35] examined the effects on executive function. We chose the random effects model due to I^2^ > 50%. The results show that combined intervention considerably enhanced executive function, with high heterogeneity (SMD = 0.03, 95% CI [-0.06-0.66], *p* = 0.10, I^2^ = 89%, Fig 4). Since the high heterogeneity, sensitivity analyses were conducted by excluding one study each time. The results indicated that there was a substantial change in the heterogeneity (I^2^ = 27%) and overall pooled effect (SMD = 0.40, 95% CI [0.25-0.56], *p* < 0.00001) when the study by Shimada et al. [28] was removed (S1 Table).

**Fig 5:**
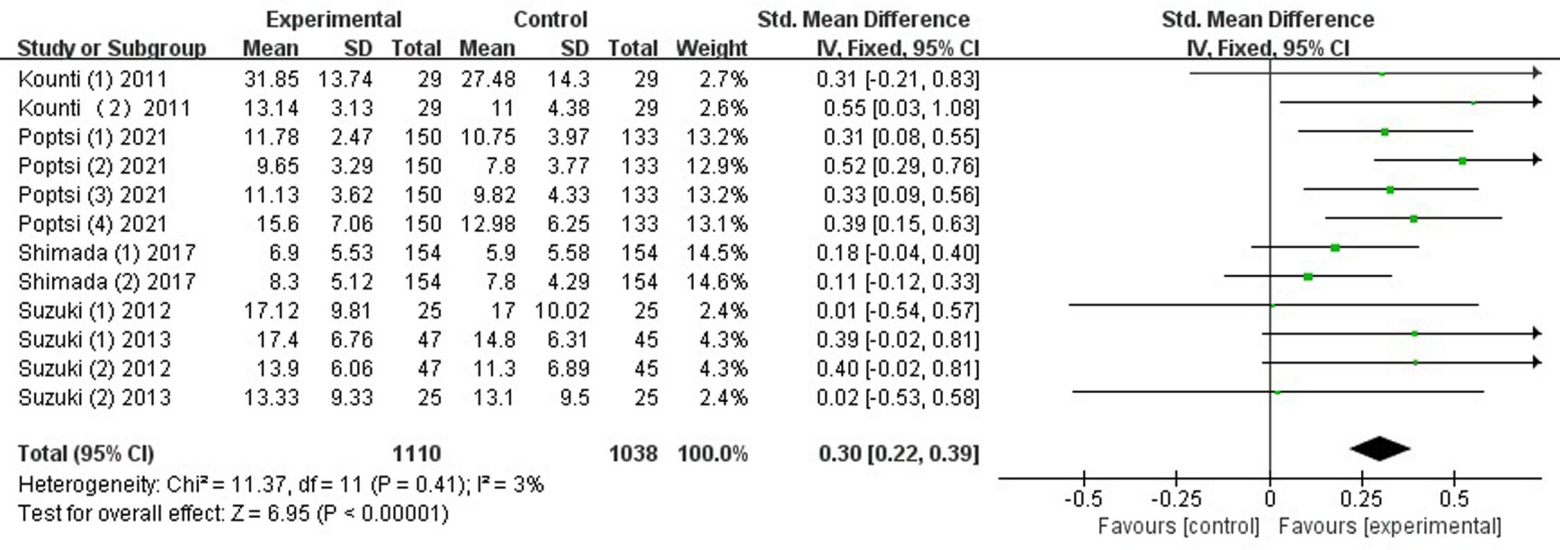
Forest plot of memory.

*Memory*. As shown in Fig 5, five studies [26–28, 32, 35] that examined the memory gains of combined intervention. The findings indicate that combined intervention greatly enhanced memory, with low heterogeneity (SMD = 0.30, 95% CI [0.22-0.39], *p* <0.00001, I^2^ = 3%).

**Fig 6:**
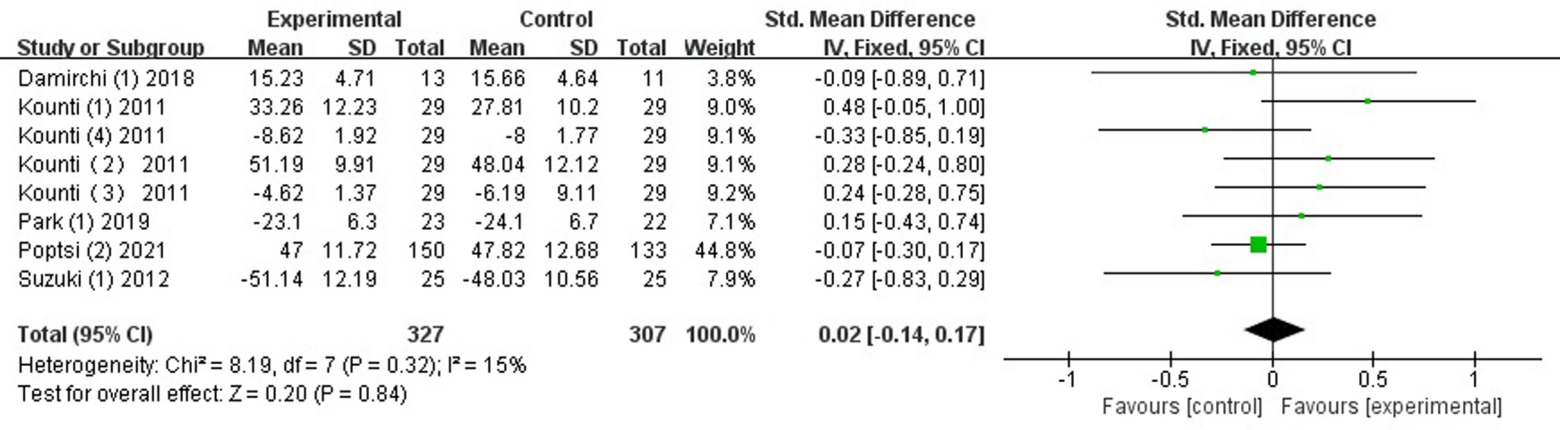
Forest plot of attention.

*Attention*. Five studies [26, 31, 32, 34, 35] evaluated the effectiveness of the combined intervention on attention. The results show that no significant improvement was observed with combined intervention on attention, with low heterogeneity (SMD = 0.02, 95% CI [-0.14-0.17], *p* = 0.84, I^2^ = 15%; Fig 6).

**Fig 7:**
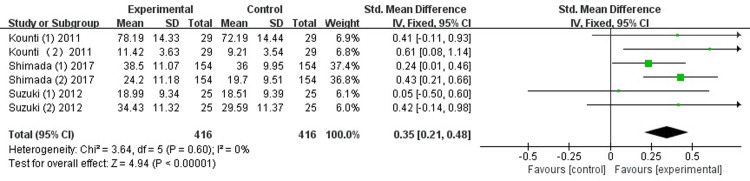
Forest plot of verbal fluency.

*Verbal fluency*. Three studies [26, 28, 32] evaluated the effects of the combined intervention on attention. The results indicated that no significant improvement was observed by combined intervention on attention, with low heterogeneity (SMD = 0.35, 95% CI [0.21-0.48], *p*<0.00001; I^2^ = 0%; Fig 7).

**Fig 8:**
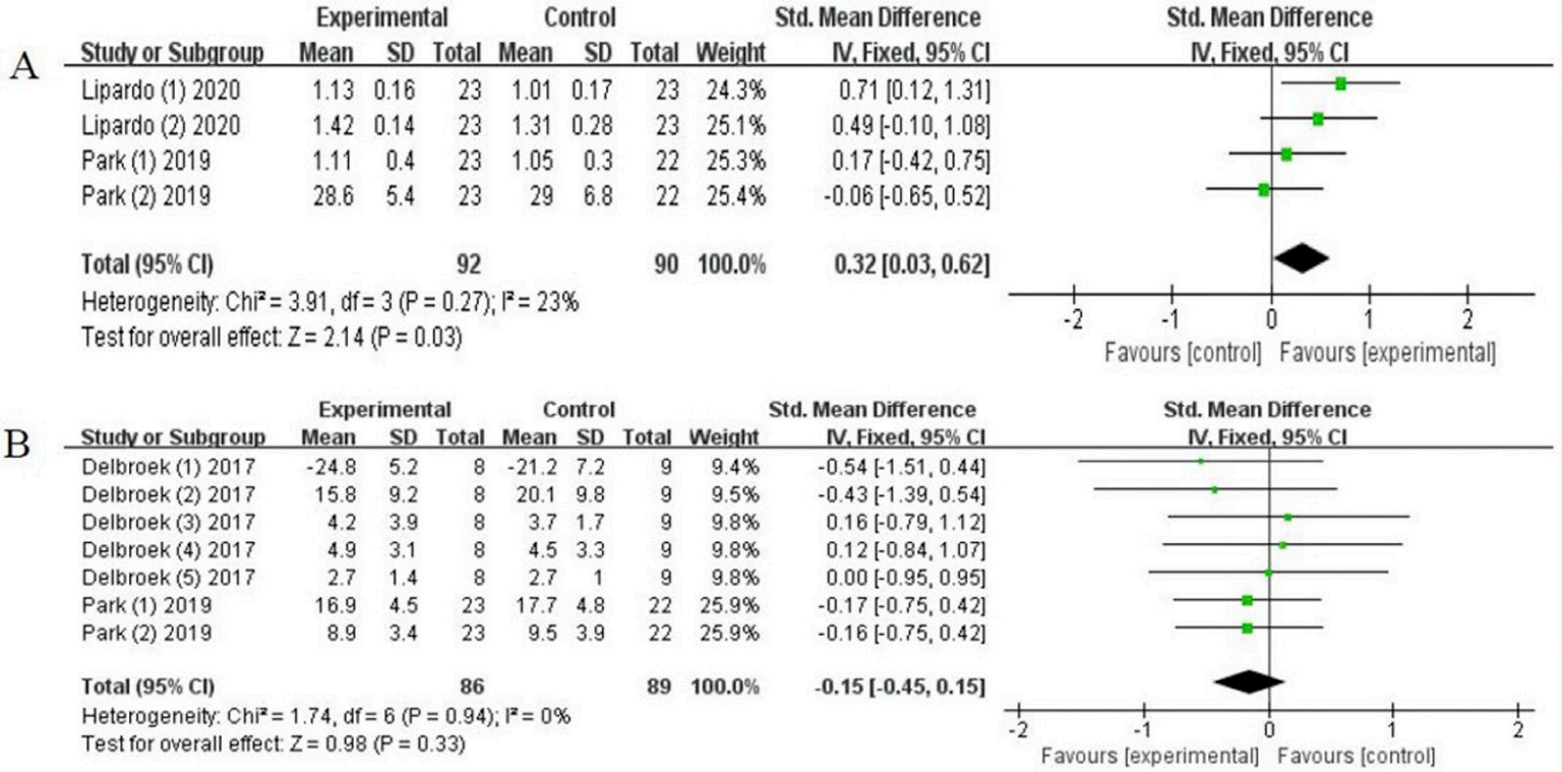
Forest plot of gait performance (A) and mobility function (B).

*Physical function*. We only assessed the effects on gait performance and mobility function due to the limited number of included studies. Two studies [31, 36] examined gait performance and the results showed a considerable improvement, with low heterogeneity (SMD = 0.32, 95% CI [0.03-0.62], *P* = 0.03; I^2^ = 23%, Fig 8A). Additionally, two studies [31, 37] assessed the efficacy of the combined intervention on mobility function. reporting no statistical difference, with low heterogeneity (SMD = -0.15, 95% CI[-0.45-0.15], *P* = 0.33; I^2^ = 0%; Fig 8B).

### Effects of combined intervention versus single exercise or cognitive intervention

**Fig 9:**
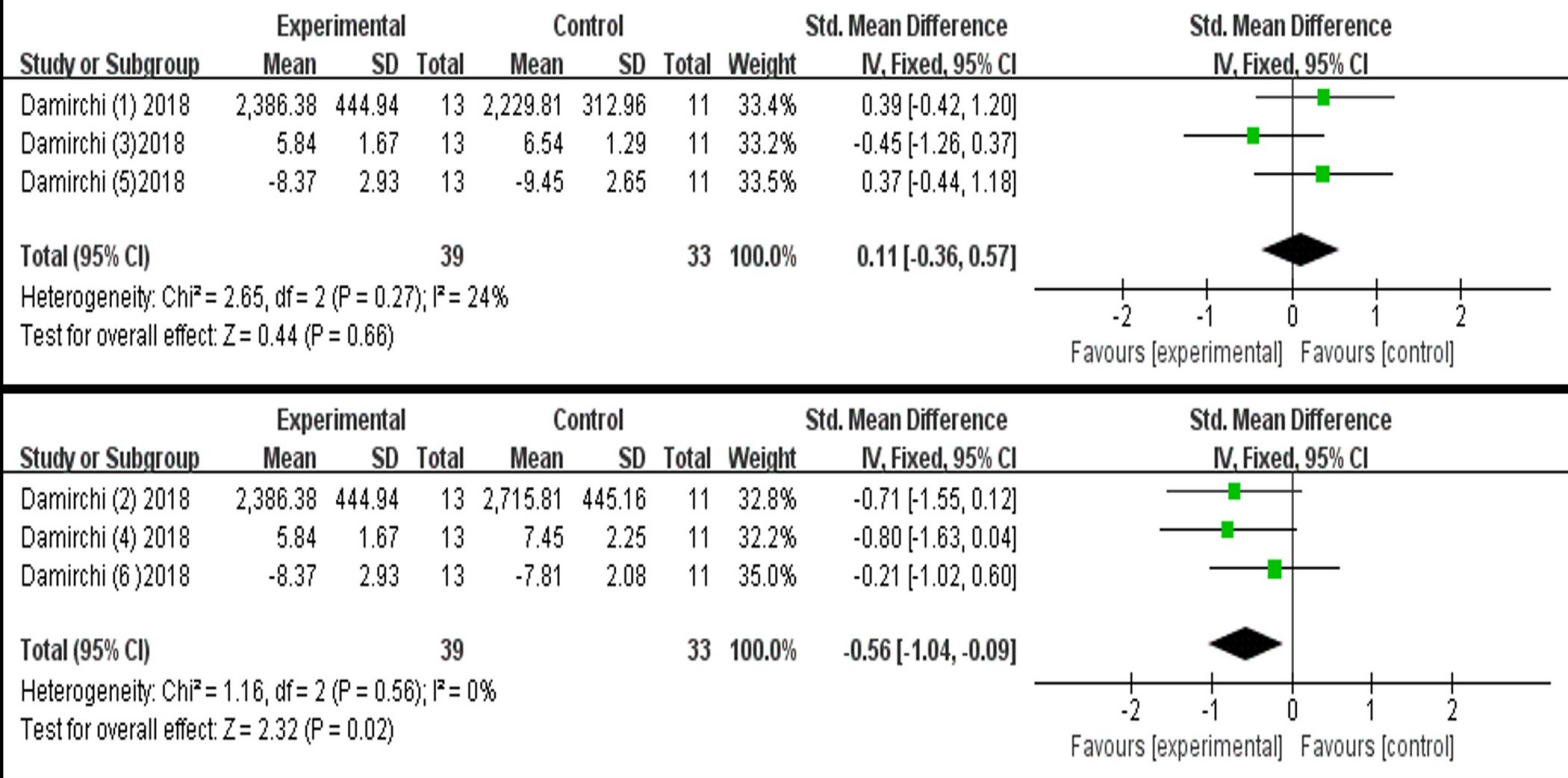
Forest plot of executive function.

Due to the limited included studies, we only examined the efficacy of the combined intervention on executive function. Specifically, one study [34] with three eligible data examined the effect of combined intervention compared to single cognitive intervention, and indicated no statistically significant, with low heterogeneity (SMD = 0.11, 95% CI [-0.36-0.57], *p* = 0.66, I^2^ = 24%, Fig 9A). Moreover, this study [34] evaluated the effectiveness of combined intervention versus single exercise intervention, showing a significant positive efficacy, with low heterogeneity (SMD = -0.56, 95% CI [-1.04 - -0.09], *p* = 0.02, I^2^ = 0%, Fig 9B).

### Effects of simultaneously versus sequentially combined intervention

**Fig 10:**
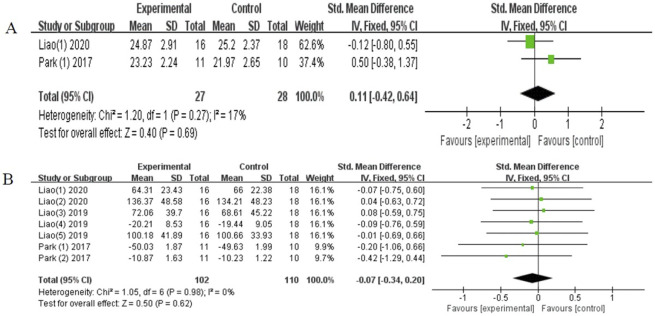
Forest plot of global cognition (A) and executive function (B).

The effectiveness of global cognition was investigated in two studies [29, 33], and the results indicated that no significant improvement, with low heterogeneity (SMD = 0.11, 95% CI [-0.42-0.64], *P* = 0.68; I^2^ = 17%, Fig 10A). Three studies [29, 31, 33] examined the effects on executive function, which indicated no statistically significant, with low heterogeneity (SMD = -0.07, 95% CI [-0.34-0.20], *p* = 0.62; I^2^ = 0%, Fig 10B).

### Publication bias

The number of articles included in this study for each outcome was fewer than 10. Consequently, it was infeasible to generate a funnel plot for the assessment of publication bias.

## Discussion

This study first examined the efficacy of combined intervention on the cognitive and physical function of older adults with MCI. The results indicated that combined intervention effectively enhanced global cognition, memory, executive function, verbal fluency, and gait versus the control group. Additionally, combined intervention demonstrated beneficial efficacy on the executive function compared to single exercise intervention. However, no statistical differences were found between simultaneously and sequentially combined intervention.

### Combined intervention versus control group on cognitive function

The results indicated that the combined intervention had a superior effect on global cognition versus the control group, which is consistent with previous research [10–12, 38]. Specifically, a meta-analysis investigated the effects of the combined intervention on cognitive performance in individuals with MCI or dementia [9]. The meta-analysis included 10 RCTs, with three trials on senior individuals with MCI displaying beneficial effects on global cognition. Additionally, another meta-analysis evaluated the combined intervention’s efficacy on global cognition in patients with MCI, which also found positive effects [12].

Regarding cognition domains, this study revealed a favorable efficacy of the combined intervention on executive function and memory while not on attention. A meta-analysis revealed that combination intervention enhanced majority of cognitive performance in seniors with MCI, but had no impact on attention, aligning with current findings [10]. Conversely, a meta-analysis found that the combined intervention had no discernible impact on executive function, attention, or memory [9]. One possible explanation is that executive and memory functions both comprise many subcomponents. Hence, diverse studies may include varying subcomponents, resulting in disparate combined results [39]. Sensitivity analysis showed reduced heterogeneity after excluding Shimada et al.’s study, possibly due to variations in cognitive rating scales across studies. Notably, Shimada et al. used the Trail-Making Test (TMT) to measure executive function, differing from the other five studies [28].

The current understanding of neural mechanisms in combined cognitive interventions is inconclusive. Animal studies suggest that physical exercise and an enriched environment induce hippocampal neurogenesis through different pathways, and the combination leads to greater benefits than either a single physical exercise or an enriched environment [40]. Furthermore, exercise and cognitive interventions may share identical or complementary roles, generating synergistic effects on cognition. Specifically, both contribute to increased cerebral blood flow, enhanced white matter connection integrity, and greater brain volume [41]. Complementary mechanisms include exercise supporting neuronal cell proliferation and division [42, 43], while cognitive intervention helps promote the survival of these newborn cells [44].

### Combined intervention versus control group on physical function

Regarding the physical function, two studies [31, 37] were included in this analysis. The present study indicated that combined intervention yields favorable effectiveness on gait performance but not on mobility, partially supporting previous findings. Ali et al. [45] conducted a meta-analysis that showed a positive impact on gait speed and balance. Another review demonstrated that combined intervention improved the mobility and gait speed of patients with MCI [46]. Possible reasons for the combined intervention improving gait but not mobility are twofold: (1) The combined intervention could improve older adults’ cognitive function, balance, and muscle strength, making it easier for them to adapt to different walking environments; (2) The interaction of cognitive intervention reduces the intensity of exercise training, which limits the growth of lower extremity muscle power. However, lower extremity muscle power plays a prominent role in improving mobility among older adults [47, 48].

### Combined intervention versus single exercise or cognitive intervention

This study indicated that combined intervention effectively enhanced executive function versus single exercise intervention, but there was no statistically significant compared to single cognitive intervention, which is partially in line with prior research [12]. Specifically, this meta-analysis reported that both had favorable benefits on executive function/attention when compared to single exercise and cognitive intervention. We speculate that the effects of cognitive intervention are generally more effective than those of exercise intervention. Thus, if the design of the combined intervention is not well-optimized, it may lead to dual-task interference, and its effects could be inferior to those of a single cognitive intervention. However, the findings should be interpreted with caution since only 6 eligible data points were included in the quantitative analysis from one study [34]. Therefore, more studies with multi-armed designs should investigate the effectiveness of the combined intervention versus single exercise and cognitive intervention.

### Simultaneously versus sequentially combined intervention

The effectiveness of simultaneously versus sequentially combined intervention remains inconclusive. Our study found no statistical significance between the two categories of combined intervention in improving global cognition and executive function. One network meta-analysis reported that sequentially combined intervention is the most effective mode for global cognition, followed by simultaneously combined intervention [15]. However, two studies have shown that simultaneously combined intervention is more effective than sequentially combined intervention [11, 49]. The possible neural mechanism for the greater cognitive gains of simultaneous training are as follows. The possible neural mechanism is related to the temporary release of neurotrophic factors after exercise intervention [50]. To reap the greatest benefits, studies have shown that physical and mental tasks should be carried out concurrently [51], with possible order effects [52].

## Limitations

Firstly, the included studies exhibit significant variability in the characteristics of the interventions, such as combination modes, intervention length, frequency, and duration. This heterogeneity may have influenced the overall effect sizes and generalizability of the findings. Secondly, the number of included studies comparing combined intervention with single exercise and cognitive intervention was limited, potentially affecting the robustness of the comparisons. Lastly, the articles published in non-English languages were excluded, potentially introducing language bias.

## Conclusions

This study, involving 12 articles assessing the effects of the combined intervention on cognitive and physical function, demonstrated that global cognition and majority of cognition domains were considerably enhanced when compared to the control group. However, the efficacy of the combined intervention versus single exercise or cognitive intervention, as well as the two different forms of combined intervention, is inconsistent. Furthermore, more well-designed studies should explore the effective and potential mechanisms of the combined intervention.

## Supporting information

S1 Appendix Search strategies. (DOCX)

S2 Appendix PRISMA 2020 checklist. (DOCX)

S1 Table Sensitivity analysis of executive function. (DOCX)
