# Supplementary material for: Cognitive and physical impact of combined exercise and cognitive intervention in older adults with mild cognitive impairment: A systematic review and meta-analysis
Source: PLoS One. 2024 Oct 3;19(10):e0308466. doi: 10.1371/journal.pone.0308466 (PMC11449338; doi:10.1371/journal.pone.0308466)
Supplement: S1 Appendix — (DOCX) [file pone.0308466.s001.docx]

**Combination Search Terms**

The databases were retrieved using the following combination methods: ((#1 AND #2) OR #3) AND #4 AND #5

#1 Cognitive intervention terms

cognitive training or cognitive function training or brain training or attention training or reasoning training or memory training or mental training or mental skills training or neurocognitive training or cognitive exercise or brain exercise or memory exercise or attention exercise or reasoning exercise or cognitive stimulation or memory stimulation or memory enhanc$ or cognitive enhanc$ or cognitive rehabilitat$ or cognitive remediation or cognitive restructur$ or cognitive activit$ or mental activit$ or brain speed training or braining processing training or mnemonic$ or method of loci or video game$ or videogame$ or computer game$ or virtual reality or cognitive intervention$ or neurocognitive intervention$ or neuropsychological intervention$ or cognitive rehabilitation or memory intervention or memory fitness or mental activity or mental stimulation or cognitive activity or attention training or executive function training or attentional control training or memory enhance$ or executive function enhancement

#2 Exercise intervention terms

exercis$ or sport$ or Physical fitness or aerobic exercis$ or aerobic train$ or aerobic fitness or aerobic program$ or resistance exercis$ or resistance train$ or anaerobic exercis$ or anaerobic train$ or resistance program$ or motor or movement or functional training or core training or acute exercise$ or isometric exercises or flexibility training or high-intensity interval training or muscle or gait or walking cardiorespiratory or cardiovascular or resistance or strength or bicycl$ or bike rid$ or bicycle rid$ or multimodal or multidomain or multicomponent or multi-modal or multi-domain or multi-component or tai chi or taiji or tai chi chuan or danc$ or physical training or physical activity or aerobic activity or strength training or balance training or Physical education and training or yoga or martial arts

#3 Combined intervention terms

multimodal or multidomain or multicomponent or multi-modal or multi-domain or multicomponent or dual task or dual-task or combined intervention or exercise combined with cognitive or cognitive combined with exercise or physical activity combined with cognitive or physical combined with cognitive or combined motor-cognitive exergame$ or active video game$ or active videogame$ or kinect or “active play” or interactive video

**#4 Aging population terms**

older adults or elder$ or senior$ or adult$ or older or ag?ing or the aged or old people or senior citizen$

**#5 MCI**

cognitive dysfunction$ OR cognitive decline OR mental deterioration$ OR mild neurocognitive disorder$ OR mild cognitive impairment$ OR mildly cognitively impaired
