# Supplementary material for: Cognitive and physical impact of combined exercise and cognitive intervention in older adults with mild cognitive impairment: A systematic review and meta-analysis
Source: PLoS One. 2024 Oct 3;19(10):e0308466. doi: 10.1371/journal.pone.0308466 (PMC11449338; doi:10.1371/journal.pone.0308466)
Supplement: S1 Table — (DOCX) [file pone.0308466.s003.docx]

**Table 2.** Sensitivity analysis of executive function.

| Study omitted | SMD | 95%CI | p | I2 (%) | p |
| --- | --- | --- | --- | --- | --- |
| Damirchi (1) 2018 | 0.25 | -0.12-0.62 | ＜0.00001 | 90 | 0.19 |
| Damirchi (2) 2018 | 0.24 | -0.13-0.61 | ＜0.00001 | 90 | 0.20 |
| Damirchi (3) 2018 | 0.27 | -0.10-0.65 | ＜0.00001 | 90 | 0.16 |
| Kounti (1) 2011 | 0.30 | -0.09-0.69 | ＜0.00001 | 90 | 0.13 |
| Park (1) 2019 | 0.32 | -0.07-0.70 | ＜0.00001 | 90 | 0.11 |
| Poptsi (2) 2021 | 0.28 | -0.12-0.68 | ＜0.00001 | 89 | 0.17 |
| Poptsi (5) 2021 | 0.29 | -0.12-0.71 | ＜0.00001 | 90 | 0.16 |
| Poptsi (6) 2021 | 0.31 | -0.11-0.74 | ＜0.00001 | 90 | 0.15 |
| Shimada (1) 2017 | 0.40 | 0.25-0.56 | 0.19 | 27 | ＜0.00001 |
| Suzuki (1) 2012 | 0.33 | -0.06-0.72 | ＜0.00001 | 90 | 0.10 |
| Suzuki (2) 2012 | 0.34 | -0.04-0.73 | ＜0.00001 | 90 | 0.08 |
